# Is the Risk of Motor Neuron Disease Increased or Decreased after Cancer? An Australian Case-Control Study

**DOI:** 10.1371/journal.pone.0103572

**Published:** 2014-07-24

**Authors:** Alex Stoyanov, Roger Pamphlett

**Affiliations:** The Stacey Motor Neuron Disease Laboratory, Department of Pathology, Sydney Medical School, The University of Sydney, Sydney, Australia; University of Oxford, United Kingdom

## Abstract

Cancer appears to be inversely associated with both Alzheimer's and Parkinson's disease. The relationship between cancer and sporadic motor neuron disease (SMND), however, remains uncertain. Most previous cancer-SMND studies have been undertaken in northern hemisphere populations. We therefore undertook a case-control study to see if a link between cancer and SMND exists in an Australian population. A questionnaire was used to compare past cancer diagnoses in 739 SMND patients and 622 controls, recruited across Australia. Odds ratios with 95% confidence intervals were calculated to look for associations between cancer and SMND. A history of cancer was not associated either positively or negatively with a risk of subsequent SMND. This result remained when age, gender, smoking status, and the four SMND diagnostic subgroups were taken into account. No association was observed between SMND and specific tumours, including melanoma, a common malignancy in Australia. In conclusion, this Australian case-control study does not support an association between a past history of cancer and the development of SMND. This suggests that some pathogenetic mechanisms, such as apoptosis, are less relevant in SMND than in other neurodegenerative diseases where negative associations with cancer have been found.

## Introduction

Uncontrolled cellular proliferation is responsible for cancer [1], in contrast to neurodegeneration, which appears to occur due to premature cell death [2]. The opposing mechanisms between these disease groups have led to the suggestion that an inverse association exists between them, with the occurrence of one conferring a decreased incidence of the other [3], [4]. Several large epidemiological studies have demonstrated that both Alzheimer's disease [5]–[8] and Parkinson's disease [9]–[12] have negative associations with cancer. On the other hand, some individual tumours may confer an increased risk of Parkinson's disease [13], [14]. Mechanisms proposed to explain these findings include common pathways of cell cycle protein dysregulation [15], [16], mitochondrial dysfunction [17], and aberrant oxygen sensing [18]. Further exploration of the relationship between neurodegenerative disease and cancer could provide information on their respective causes and assist in the development of new therapies.

Motor neuron disease (MND) is a neurodegenerative disorder that causes progressive muscle weakness, with an average survival of 3–5 years [19]. Early case studies reported a positive association between cancer and MND, in particular breast cancer, but these were based on small patient numbers [20]–[24]. More recently, five large epidemiological studies found no significant association between cancer and MND in northern hemisphere populations [25]–[29]. However, they did suggest the possibility of an association between two specific tumours and MND, i.e., an increased incidence of MND after melanoma [25], [27] and a decreased incidence after prostate cancer [26], [27]. Some limitations of these studies included the use of non-population controls [26] and the use of mortality rather than incidence data to diagnose MND [25], [27]. Two studies used hospital and government registries to identify MND cases [28], [29], and therefore could not include potentially confounding factors such as smoking.

The present study aimed to see if the associations between MND and cancer found in northern hemisphere countries could be replicated in a southern hemisphere population. This strategy has been used before in other epidemiological studies of MND, and have yielded different results to northern hemisphere studies, for example, in relation to smoking as a risk factor for MND [30]. Furthermore, Australia has the highest rate of melanoma in the world [31], and is well placed to determine whether the previous reports of an increased risk of MND after melanoma can be replicated. A questionnaire-based case-control study of previous cancer in an Australian MND population was therefore undertaken. The case-control design had the advantage of enabling potentially-confounding variables such as smoking to be taken into account.

## Methods

### Ethics statement

All participants gave written informed consent for the use of their clinical and environmental data. The collection of data was approved by the Human Research Ethics Committee of the Sydney South West Area Health Service.

### Study participants

Participants were selected from eligible individuals who submitted self-completed questionnaires to the Australian Motor Neuron Disease DNA Bank, centred at the University of Sydney. The DNA Bank collected clinical, demographic and environmental data from patients with sporadic MND (SMND) and controls (individuals without SMND), recruited via MND Associations in each state of Australia. The duration of data collection was between 2000 and 2011. Inclusion criteria for this study included being a white Australian resident over the age of 26 years.

Cases were those individuals diagnosed with SMND for whom clinical notes and special investigations were available from treating neurologists. Patients with SMND were classified as having SALS if they fulfilled the probable or definite revised El Escorial criteria for ALS (with both upper and lower motor neuron signs) [32]. Patients with a progressive lower motor neuron disorder with no upper motor neuron signs were classified as having sporadic progressive muscular atrophy (SPMA); those with a progressive upper motor neuron disorder without lower motor neuron signs were classified as having sporadic primary lateral sclerosis (SPLS); and those who had a progressive upper and lower motor neuron disorder involving only the bulbar muscles were classified as having sporadic progressive bulbar palsy (SPBP). Controls consisted of individuals without SMND who were partners or friends of SMND patients, or community volunteers.

### Identification of cancers

Identification of cancer was based on individuals' responses to the questions asking “List any significant past medical illness or health problems” and “List any previous injuries and/or surgeries”. It was assumed a diagnosis of cancer was a significant enough event in the life of participants for almost full reporting. Keywords used to identify malignancies were “cancer”, “cancerous tumour”, “malignant tumour”, “malignancy” or specific individual malignancies such as “melanoma”. In addition, reporting a surgery or procedure that would be undertaken exclusively for a particular cancer was included as a cancer item (e.g., patients having undergone a radical prostatectomy were assumed to have had prostate cancer). No benign tumours were classified as cancers. For all cancers identified, the following details were noted: (1) year of diagnosis; (2) tumour type (e.g., carcinoma or sarcoma); and (3) the organ affected.

### Diligence in answering the questionnaire

The presumably non-relevant questions “Have you ever had a pet?” and “If yes, list the types of pets you have had” were included to compare the diligence of the SMND and control groups in answering the questionnaire.

### Statistical analysis

Data were analysed using SPSS v20. Chi-square and Fisher's exact tests from contingency tables were used to determine odd ratios (ORs) with 95% confidence intervals (CIs) for developing SMND, in those with and without cancer. Unconditional logistic regression analyses were used to correct for confounding of covariates.

Unconditional logistic regression gives inaccurate estimates if a covariate has a strong association with both the dependent and independent variable [33]. Multiple regression analyses showed significant colinearity between age and both SMND and cancer incidence, so age was not corrected as a covariate in the analysis. Due a statistically significant difference in average age between SMND and control females initially, a nested cohort was created in which female controls were randomly matched to a female SMND patient within a 5-year age range at a 1∶1 ratio. This resulted in almost identical mean ages between the two cohorts for both males and females (Table 1), and allowed age to be excluded from the logistic regression analysis.

**Table 1:** Gender, age and smoking status in SMND and control individuals.

	SMND *N* (%) {SD}	Control *N* (%) {SD}
*Males*		
Total	488	371
Age range (years)	30–90	27–94
Mean age (years)	62.6 {11.2}	62.4 {12. 6}
Number of smokers	298 (61)	222 (60)
Mean pack years smoked	14.4 {20.9}	15.6 {22.6}
*Females*		
Total	251	251
Age range (years)	27–82	35–86
Mean age (years)	62.8 {9.8}	62.6 {9.9}
Number of smokers	103 (41)	105 (42)
Mean pack years smoked	7.7 {14.9}	7.0 {14.2}

*N*: number, SD: standard deviation.

The potential covariates contributing to the risk of cancer were considered to be gender and smoking history. Subgroup analyses were undertaken in: (1) 10-year age groups from 41 to 90 years (this excluded 68 participants aged below 40 years and two aged over 90 years); (2) smokers and non-smokers; (3) different types of tumours; and (4) the four SMND clinical subgroups. Within the smoker and non-smoker subgroups, smoking-related cancers (of the oral cavity, throat, oesophagus, stomach, pancreas, lung, cervix, bladder, kidney and colon) and non-smoking-related cancers [34] were considered separately. Where numbers were insufficient for logistic regression (<5 individuals per cell), contingency tables were used to calculate ORs, 95% CIs and Fisher's exact p-values [35].

## Results

### Cases and controls

739 (488 male, 251 female) patients made up the SMND group (Table 1). Of these, 78% had SALS, 14% had SPMA, 5% had SPLS and 3% had SPBP. 622 (371 male, 251 female) individuals comprised the control group; of these, 54% were partners of SMND patients, 28% were community volunteers, and 18% were friends of SMND patients.

The SMND group had a higher proportion of males (66%) compared to the controls (60%) but both had almost identical mean ages (Table 1). The proportion of SMND males and females who smoked, and the average pack years smoked, was similar to controls (Table 1).

### Cancer risk in SMND patients and controls

#### Males and females combined

Sixty-four (8.7%) of the SMND patients had a history of cancer, compared to 59 (9.5%) of the controls. A history of cancer did not therefore significantly alter an individual's risk of developing SMND (Table 2).

**Table 2:** Comparison of cancer in SMND patients and controls.

	SMND *N* (%)	Control *N* (%)	OR (95% CI)	p-value
*Males and females*				
Total	739	622		
Cancer	64 (8.7)	59 (9.5)	0.90 (0.62–1.32)	0.58
Non-cancer	675	563		
*Males*				
Total	488	371		
Cancer	46 (9.5)	37 (10.0)	0.96 (0.61–1.52)	0.86
Non-cancer	442	334		
*Females*				
Total	251	251		
Cancer	18 (7.2)	22 (8.8)	0.79 (0.42–1.53)	0.49
Non-cancer	233	229		

Odds ratios (ORs) adjusted for gender and smoking.

CI: confidence interval, *N*: number.

#### Males

9.5% of SMND and 10.0% of control men had a history of cancer. The proportion of men with a previous cancer did not differ significantly between the groups (Table 2).

#### Females

7.2% of SMND and 8.8% of control females had a history of cancer. The proportion of females with a history of cancer did not differ significantly between the groups (Table 2).

### Cancer and SMND within age groups

No significant association was identified between a history of cancer and SMND in any 10-year age subgroups from 41–90 years (Table 3). The prevalence of both cancer and SMND increased with age (χ^2^ trend, p = 0.037). However, the prevalence of cancer between the two groups did not change with age (χ^2^ trend, p = 0.19).

**Table 3:** Comparison of cancer in SMND patients and controls in 10-year age groups.

Age groups	SMND *N* (%)	Control *N* (%)	OR (95% CI)	p-value
*41–50 y*				
Total	77	80		
Cancer	4 (5.2)	5 (6.3)	0.80 (0.20–3.17)	0.75
*51–60 y*				
Total	200	142		
Cancer	7 (3.5)	7 (4.9)	0.63 (0.22–1.85)	0.40
*61–70 y*				
Total	268	221		
Cancer	26 (9.7)	19 (8.6)	1.11 (0.60–2.07)	0.74
*71–80 y*				
Total	146	138		
Cancer	20 (13.7)	24 (17.4)	0.79 (0.41–1.54)	0.50
*81–90 y*				
Total	26	20		
Cancer	7 (26.9)	4 (20.0)	1.45 (0.36–5.97)	0.59

Odds ratios (ORs) adjusted for gender and smoking.

CI: confidence interval, *N*: number.

### Cancer and SMND in smokers

728 participants had a history of smoking, with an average of 11.7 pack years (SD 19.2 pack years) per participant, while the remaining 633 had no history of smoking (Table 4). Of the smokers, 54% of the SMND patients smoked compared to 53% of controls. Unexpectedly, cancer prevalence was similar in smokers and non-smokers: 8.9% of smokers had a history of cancer compared to 9.2% of non-smokers. A history of cancer did not alter future SMND risk in either smokers or non-smokers (Table 4). When cancers were divided into smoking and non-smoking-related cancers, no association between SMND and either cancer group was found (Table 4).

**Table 4:** Comparison of smoking and non-smoking-related cancers in SMND patients and controls.

	SMND *N* (%)	Control *N* (%)	OR (95% CI)	p-value
*Smokers*				
Total	401	327		
Any cancer	33 (8.2)	32 (9.8)	0.85 (0.50–1.42)	0.53
Smoking-related cancer	10 (2.5)	8 (2.4)	1.01 (0.39–2.61)	0.99
Non-smoking-related cancer	23 (5.7)	24 (7.3)	0.80 (0.44–1.46)	0.52
No cancer	368	295		
*Non-smokers*				
Total	338	295		
Any cancer	31 (9.2)	27 (9.2)	0.98 (0.57–1.69)	0.94
Smoking-related cancer	8 (2.4)	7 (2.4)	0.96 (0.35–2.71)	0.95
Non-smoking-related cancer	23 (6.8)	20 (6.8)	0.99 (0.53–1.84)	0.97
No cancer	307	268		

Odds ratios (ORs) adjusted for gender and smoking.

CI: confidence interval, *N*: number.

### SMND and tumour subgroups

The most common malignancies in the cohort were melanoma, prostate cancer, non-melanoma skin cancer, and colorectal cancer (Table 5). Several of the tumours (including lung, soft tissue, testicular and uterine tumours) had no cases in one of the groups, hence an odds ratio was unable to be calculated. Since this was a case-control study, cancers with low survival rates were almost non-existent so analysis of aggressive, short-survival tumours was not possible. Only one case of lung cancer was identified, while no brain, pancreatic or other high grade tumours were present.

**Table 5:** Comparison of cancer subgroups in SMND patients and controls.

	SMND *N*	Control *N*	OR (95% CI)	p-value
Total	739	622		
Cancer	64	59	0.90 (0.62–1.32)	0.58
*Tumour subgroup*				
Bladder	2	2	0.84 (0.12–5.99)	1.00
Breast	7	5	1.40 (0.44–4.50)	0.57
Colorectal	9	7	1.05 (0.39–2.83)	0.93
GIT other	1	1	0.84 (0.06–13.48)	1.00
Head and neck	3	4	0.63 (0.14–2.83)	0.71
Haematopoietic	4	2	1.69 (0.31–9.24)	0.69
Renal	3	2	1.26 (0.21–7.59)	1.00
Lung	1	0	NA	1.00
Prostate	8	13	0.47 (0.19–1.13)	0.09
Melanoma	12	10	0.97 (0.42–2.30)	0.97
N-M skin cancer	11	6	1.53 (0.57–4.21)	0.40
Soft tissue	0	1	NA	0.46
Testes	2	0	NA	0.51
CUP	1	3	0.28 (0.03–2.70)	0.34
Uterine	0	3	NA	0.25

Odds ratios (ORs) adjusted for age, gender and smoking with logistic regression where case numbers were ≥5, and with contingency tables when case numbers were <5. Where one cell contained no values, no ORs were calculated but p-values were calculated with Fischer's exact test.

CI: confidence interval, CUP: cancer of unknown primary, GIT: gastrointestinal tract, *N*: number, NA: not applicable, N-M: non-melanoma.

Individuals with a history of cancer of unknown primary and males with prostate cancer tended to have a lower risk of SMND (Table 5), but these did not reach statistical significance. The rates of melanoma did not differ significantly between SMND and control groups (Table 5). No association between any other tumour subgroups and SMND was apparent.

### Cancer in SMND clinical subgroups

8.8% of individuals within the SALS subgroup had a history of cancer compared to 7.8% in the SPMA, 21.0% in the SPBP, and 2.7% in the SPLS subgroups (Table 6). Despite the apparently increased history of cancer in the SPBP group, contingency table analysis showed no difference in cancer frequency between the four groups (p = 0.15). When individual SMND subgroups were compared to total numbers of controls, a history of cancer did not significantly alter SALS or SPMA risk (Table 6). Individuals with cancer tended to be at increased risk of SPBP and decreased risk of SPLS, but these results did not reach statistical significance (Table 6).

**Table 6:** Cancer in SMND clinical subgroups compared to controls.

SMND subgroup	Total *N*	Cancer *N* (%)	OR (95% CI)	p-value
ALS	580	51 (8.8)	0.91 (0.62–1.36)	0.66
PMA	103	8 (7.8)	0.78 (0.36–1.70)	0.53
PBP	19	4 (21.0)	2.55 (0.82–7.92)	0.11
PLS	37	1 (2.7)	0.27 (0.04–1.97)	0.24

Odds ratios (ORs) adjusted for gender and smoking using logistic regression where case numbers were ≥5. Where cases were <5 in number, ORs were calculated using contingency tables.

CI: confidence interval, *N*: number, ALS: amyotrophic lateral sclerosis, PMA: progressive muscular atrophy, PBP: progressive bulbar palsy, PLS: primary lateral sclerosis.

### Diligence in answering the questionnaire

Of the controls, 99.7% answered the question on having pets, and 83% listed the pets they had. In comparison, 99.6% of SMND patients answered the question on having pets, and 82% listed the types they had (both insignificant differences).

## Discussion

This Australian case-control study found no association between a history of cancer and the later onset of SMND. A history of cancer did not increase or decrease the incidence of any SMND subgroups, including the most common subgroup, SALS. No cancer-SMND associations were present when age groups, gender, and smoker status were taken into account. In addition, no association with SMND was noted for either smoking or non-smoking related cancers. No individual tumours, including melanoma and prostate cancer, altered the risk of SMND.

The present study has several advantages over many of the previous studies of cancer and MND. This is largest cancer-MND case-control study to date, and the use of a questionnaire allowed the collection of lifestyle data including smoking history. No reliance on registry or *post mortem* data for diagnosis was needed, since all SMND patients had the diagnosis made by a neurologist with supporting clinical notes and investigations. This ensured SMND diagnosis was accurate and that clinical information was available to identify SMND subgroups. Finally, the use of partner and friend controls, with similar social and environmental lifestyles, reduced the chance of type I statistical errors [36].

This cancer-SMND study is the first to control for smoking status. Smoking is a well-established risk factor for a large number of cancers [34] and may also increase the risk of developing SALS [37], [38], though not all studies have found this [30]. Hence, the relationship between cancer and SMND may have been confounded by smoking status in previous studies.

This is the only overall cancer-SMND study to be conducted in the southern hemisphere; one joint USA-Australian study has looked specifically at melanoma and MND [39]. The findings of our study are consistent with most previous epidemiological studies of cancer and SMND in northern hemisphere populations. Two studies based on the SEER program of the US National Cancer Institute and death certificates found no association between cancer incidence and SALS mortality [25], [29]. Because of their use of mortality data, these studies were only able to identify SALS cases with 70–90% accuracy [40]. Other studies investigating SALS incidence based on registries rather than mortality have also shown no differences in the incidence of cancer between SALS patients and controls [26], [28], [29]. The null association across genders, age groups and between smoking and non-smoking-related cancers in a Swedish case-control study [28] is consistent with our findings.

Previous studies indicating lower cancer rates in Alzheimer's and Parkinson's diseases suggested that these neurodegenerative disorders are pro-apoptotic states, while cancer is anti-apoptotic in nature. The role of apoptosis in MND is unclear, however, and the rapid morphological changes of apoptosis make it difficult to detect in *post mortem* tissue of a disease that progresses over years [41]. Alterations in the expression of regulatory apoptotic molecules including Bax, Bak, capsases and p53 have been observed in MND animal models [42], [43] and human CNS samples have suggested that apoptosis is involved in MND [44]. However, the null relationship between cancer and MND presented here supports the opposing concept that apoptosis is not a major pathological mechanism of motor neuron degeneration in MND [45]–[47].

Of interest is the reported association between MND and specific tumours, in particular melanoma [29], [39]. We found a history of melanoma was not associated with SMND in an Australian population. Our findings are consistent with register studies from Sweden [28] and the USA [29] that found no melanoma-SMND association. In contrast, a joint USA-Australian study of post-melanoma survival found increased mortality due to MND and Parkinson's disease [39], and survivors of melanoma were also found to have an increased risk of MND in another USA population [27]. However, these two studies could not define the temporal relationship between MND and melanoma diagnosis. This raises the possibility that the positive associations may have been due to a short-term increase in medical surveillance immediately after melanoma diagnosis. While a detailed temporal analysis was not possible in our study, all but one of our SMND diagnoses were made greater than five years after melanoma diagnosis, so here any association would be long term. An association between neurodegenerative disorders and melanoma does, however, remain plausible. Riluzole, a drug used in the treatment of ALS, has been proposed as an anti-melanoma agent [48], suggesting a biochemical link between the two diseases. An increased risk of Parkinson's disease after melanoma diagnosis has been described [49], [50] and is thought to occur due to increased alpha-synuclein expression in melanocytic lesions [51]–[53] that may interact with cell cycle regulators [54]. Alpha-synuclein has been detected in MND animal models [55] and human spinal cords [56], making a similar mechanism in SMND possible. The relationship between MND and melanoma therefore requires greater exploration, with larger population sizes and accurate analysis of temporal relationships.

Previous studies have reported a negative association between prostate cancer and MND [26], [27], whereas our study found only a trend towards a negative association. Assessments of this particular association are difficult due to wide variations in methods of prostate cancer detection, the range of malignancy in prostate neoplasms, and the rising detection of indolent prostate cancers via prostate specific antigen measurement, which would create changing detection rates of prostate cancer over follow-up periods [57].

Limitations of the present study are: (1) The small numbers in some subgroups, particularly of individual tumours. (2) The risk of recall bias with any questionnaire-based approach, though both SMND and control individuals appeared to answer the questionnaire with similar diligence. (3) Some participants may not have considered certain malignancies (such as non-melanoma skin cancers, which are common in Australia) to be clinically significant enough to list in their past medical history. (4) A number of respondents did not enter the year their cancer was diagnosed, which made a temporal analysis between cancer diagnosis and MND unfeasible.

In conclusion, this case-control study has shown no significant association, either positive or negative, between a history of cancer and the occurrence of SMND in an Australian population. This finding remained when adjusted for age, gender and smoking status. In contrast to previous studies, no specific individual malignancies appeared to be associated with a diagnosis of SMND, including melanoma and prostate cancer. The present findings support increasing evidence that cancer is not inversely associated with SMND. This suggests that pathogenetic mechanisms that can be expected to protect against cancer, in particular apoptosis, may not be primary pathological mechanisms in SMND. Our findings also imply that the pathogenesis of cell damage in SMND is likely to be different from that of Alzheimer's and Parkinson's diseases, where an inverse relationship between cancer and disease onset appears to be likely.
